# DEMENTIA CAREGIVING NEGATIVELY AFFECTS THE HEALTH OF CAREGIVER AND CARE RECIPIENT. CAREGIVING TRANSITIONS STUDY

**DOI:** 10.1093/geroni/igz038.801

**Published:** 2019-11-08

**Authors:** Orla C Sheehan, William E Haley, Virginia Howard, Jin Huang, J David Rhodes, David L Roth

**Affiliations:** 1 Center on Aging and Health, Johns Hopkins University School of Medicine, Baltimore, Maryland, United States; 2 University of South Florida, Tampa, Florida, United States; 3 University of Alabama at Birmingham, Birmingham, Alabama, United States; 4 Johns Hopkins University School of Medicine, Baltimore, Maryland, United States; 5 School of Public Health, Department of Biostatistics, Birmingham, Alabama, United States

## Abstract

Dementia is one of the most common reasons for needing a caregiver (CG). Few studies have compared dementia and non-dementia caregivers who have transitioned into family caregiving roles. Participants in the REasons for Geographic and Racial Differences in Stroke (REGARDS) study who transitioned into a significant caregiving role were recruited to participate in the Caregiving Transitions Study (CTS). Of 11,483 REGARDS participants who were not caregivers at baseline, 1229 (11%) transitioned into a family caregiving role. Eligibility criteria were met by 251 and they were enrolled along with 251 demographically-matched noncaregiving controls. Enrolled caregivers are 65% female; 36% African American; 71.8 + 8.1 years of age; caring for a spouse/partner (51%), parent (25%), or another person (24%). 47% are caring for a person with dementia. Dementia CGs provide more hours of care per day (9.3 hours versus 6.7 hours), report being under more stress and twice as much strain as non-dementia CGs (p<0.03 for all). They feel more burdened by the care recipient’s treatment (p=0.01) and report that the burden leads to delays in the care recipient receiving medical care (p<0.007). Dementia CGs are more than twice as likely as non-caregivers to report that their caregiving makes them worse at taking care of their own health (33.9% versus 15.4%, p=0.003). This prospective, population-based study confirms previous cross-sectional findings from convenience samples on the greater care burden experienced by dementia caregivers and extends this work to new measures of treatment burden and treatment delay.

